# Book Reviews

**Published:** 1895-07

**Authors:** 


### *BOOK REVIEWS.*

---

System of Surgery. Edited by Frederic S. Dennis, M.D., Professor of the Principles and Practice of Surgery, Bellevue Hospital Medical College, Surgeon to Bellevue and St. Vincent Hospitals, etc.; Assisted by John S. Billings, M.D., LL.D., Deputy Surgeon-General, United States Army. Four volumes. Volume I. Profusely Illustrated. Lea Bros. & Co., 706–708 Sansom St., Philadelphia.

This is the first volume of what promises to be a most important work on surgery. Four volumes will constitute the series. It will be distinctly an American work, and among those contributing to its pages are some of the best known American surgeons and teachers.

The first volume very properly begins with a most interesting and valuable *résumé* of the "History and Literature of Surgery," by Dr. Billings, which is well worth careful reading. Dr. W. T. Councilman, Professor of Pathology at Harvard, next describes "Surgical Pathology, Including Inflammation and the Repair of Wounds." The "General Bacteriology of Surgical Infections" is described by Professor W. H. Welch, of Johns Hopkins University. This section is very full and complete. Professor Chas. B. Nancrede, of the University of Michigan, discusses the "Symptoms, Diagnosis, and Treatment of Inflammation, Abscess, Ulcer, and Gangrene." "Septicemia, Pyemia, and Poisoned Wounds" are described by Professor Wm. H. Carmalt, of Yale University; "Traumatic Fever, Erysipelas, and Tetanus," by Professor J. Collins Warren, of Harvard; "Rabies, Hydrophobia," by Professor H. M. Biggs, of Bellevue Medical College and Hospital; "Gunshot Wounds," by Professor P. S. Connor, of the Medical College of Ohio; "Fractures and Dislocations," by the Editor, Professor Dennis; "Anesthesia," by Professor H. C. Wood, of the University of Pennsylvania; "The Technique of Aseptic and Antiseptic Surgery," by Professor A. G. Gerster, of the N. Y. Polyclinic; and "Operative Surgery," by Professor Stephen Smith, of the University of the City of New York.

It is hardly necessary to add that the subjects discussed in this volume have been presented by these gifted authors in a thoroughly complete and scientific manner, leaving little to be desired. The discussion on anesthesia is the most unsatisfactory and incomplete. For instance, not one word is said of local anesthesia by cocaine. Other important matter is omitted also. We note some omissions also in the section on Fractures and Dislocations. But these objections are slight in comparison with the commendation which the book as a whole deserves. We think the complete work will be generally accepted by the profession as the most recent and accurate exponent of modern surgery.

---

PATHOLOGY AND TREATMENT OF DISEASES OF THE SKIN. *For Practitioners and Students.* By Dr. Moriz Kaposi, Professor of Dermatology and Syphilis, and Chief of the Clinic and Division for Skin Diseases in the Vienna University. Translation of the last German edition. New York: William Wood and Company. Bound in extra muslin at $5.50 per copy, and in leather at $6.50 per copy.

This is a translation of the fourth German edition of Kaposi's work, the translation having been made under the supervision of Dr. Jas. C. Johnston. The preface is written by Dr. Geo. Henry Fox. It is one of the most complete of the many books which have been written upon the subject of dermatology. Kaposi, as the successor and son-in-law of "The Father of Dermatology," the elder Hebra, and as an able worker in the field, is eminently fitted as the exponent of the Vienna "school" of dermatology. The work begun by Hebra has been brilliantly carried on by Kaposi, and he brings to the production of this book a richness of knowledge and a fullness of experience scarcely to be equaled in any other writer. A most erudite history of dermatology is given in the beginning, together with a description of the pathological position of dermatology. The book is divided into lectures, first "general," then "special."

The pathology of skin diseases is very completely described.

Many old ideas regarding the cure of skin diseases too suddenly are proven erroneous. As the representative of the Vienna "school," he writes upon many subjects in a controversial manner, his views regarding various diseases being different from those of many dermatologists.

The whole work is deep and learned, but for the most part it seems clear and practical.

Illustrations are profuse and show everything from normal anatomy of the skin through the pathology and including the animal parasites.

The work deserves a high popularity among those who are interested in the subject. M. B. H.

---

SUGGESTIVE THERAPEUTICS IN PSYCHOPATHIA SEXUALIS; with Especial Reference to Contrary Sexual Instinct. By Dr. A. Von Schrenck-Notzing (Munich, Germany). Authorized translation from the German by Charles Gilbert Chaddock, M.D., Professor of Diseases of the Nervous System, Marion-Sims College of Medicine, St. Louis, etc. One volume, Royal Octavo, 325 pages. Extra Cloth, $2.50 net. Sold only by subscription to the medical profession exclusively. Philadelphia. The F. A. Davis Co., Publishers, 1914 and 1916 Cherry street.

This volume is a suitable companion and sequel to Krafft-Ebing's Psychopathia Sexualis. As may be judged from the title, it relates especially to *suggestion* as a curative measure in cases of perverted sexual instinct. Certainly in the hands of Dr. von Schrenck-Notzing hypnotic suggestion has made cures of what appears to have been vile and desperate cases. This work and its companion volume will be useful in their way, having more especially a medico-legal value. The present volume may be commended for the description of the suggestive method of treatment, and also for the interesting chapter relating to the history of the development of the contrary sexual feeling.

NOTES ON THE NEWER REMEDIES. Their Therapeutic Applications and Modes of Administration. By David Cerna, M.D., Ph.D., Demonstrator of Physiology in the Medical Department of the University of Texas, formerly Demonstrator of Experimental Therapeutics in the University of Pennsylvania, etc. Second edition. Enlarged and revised. Price, $1.25. W. B. Saunders, 925 Walnut street, Philadelphia.

The object of these "notes" has been to supply the practitioner with reliable information upon the "therapeutic applications of the newer remedies, especially of those whose usefulness has been more or less ascertained by clinical investigation." The literature of new remedies with which doctors are gratuitously supplied by manufacturing chemists is certainly not to be relied upon. This little volume will be found to be a fair and unbiased statement of the properties and therapeutic value of the recent remedial agents. This book will be found very convenient and useful to any one who wishes to keep thoroughly abreast of the times in the matter of the latest drugs and synthetic compounds.

---

SYLLABUS OF GYNECOLOGY. By J. W. Long, M.D., Richmond, Va., Professor of Gynecology and Pediatrics in the Medical College of Virginia, etc. W. B. Saunders, Publisher, 925 Walnut street, Philadelphia.

This syllabus is based upon the *American Text-book of Gynecology*. It has been written with a threefold object: "first, to be used as lecture notes; second, to enable the student more intelligently to follow and remember the lectures; and finally as a convenient reference for practitioners." Numerous blank leaves are inserted for additional notes. It is a complete outline of the usual subjects embraced in a lecture course on gynecology, and will be found convenient by both lecturer and student.

---

HEALTH, SANITATION, AND CLIMATOLOGY OF THE SOUTHERN STATES. A pictorial magazine of thirty pages. Subscription price, $1 per annum. Edited and published quarterly by Walter C. Murphy, M.D., 932 K street, N. W., Washington, D. C.

"The object of this journal is to collect, compile, edit, and aid in the dissemination of information on hygiene, health, medical mineral waters, sanitary science, and medical climatology."

The first numbers of this new journal are very creditable. The enterprise is well worthy of generous encouragement.

---

### LIPPINCOTT'S MAGAZINE FOR JULY, 1895.

The complete novel in the July issue of *Lippincott's* is "A Social Highwayman," by Elizabeth Phipps Train, author of "The Autobiography of a Professional Beauty." It is a tale of New York society, with a hero in whom accomplishments and virtues were incongruously joined with highly objectionable habits—a sort of urban and modernized Robin Hood.

Francis Lynde furnishes a tale of the West, "The Strike in Piñon Gulch"; Will N. Harben one of the South, "Matt Digby's Meddling," and Lieut. Charles Dudley Rhodes one of the army, "The Recall of Flathers." Yet shorter stories are "McGheoghan's Lapse," by the late Professor Willis Chamberlin, and "From Four to Five," by C. K. E.

Charles Morris gives an account of "The Railroad Invasion of Asia"; J. Kumpéi Matumoto explains the mysteries of "The Tea Ceremony of Japan," and Alvin F. Sanborn describes "The Great Market of Paris."

"The whole duty of Woman, as understood by Man in the Fourteenth Century," is an interesting paper by Emily B. Stone, with quotations, which read somewhat jocosely now, from documents of that age. As a pendant to this, Professor H. H. Boyesen has a lively and extremely modern article on "The New Womanhood."

"Our National Extravagance" is exposed and deplored by Frances Courtney Baylor. Under the heading, "Fact in Fiction," Frederic M. Bird gives reasons for thinking that the two are best apart, verisimilitude, not verity, being what is wanted in fictitious narratives.

The poetry of the number, besides couplets by Grace F. Pennypacker and Calvin Dill Wilson, is by Celia A. Hayward, Juliet V. Strauss, and Clinton Scollard.

